# Evaluation of log odds of positive lymph nodes in predicting the survival of patients with non-small cell lung cancer treated with neoadjuvant therapy and surgery: a SEER cohort-based study

**DOI:** 10.1186/s12885-022-09908-3

**Published:** 2022-07-20

**Authors:** Qing Wang, Suyu Wang, Zhiyong Sun, Min Cao, Xiaojing Zhao

**Affiliations:** 1 Department of Thoracic Surgery, Renji Hospital, Shanghai Jiao Tong University School of Medicine, 160 Pujian Road, Shanghai, 200127 China; 2 Department of Cardiothoracic Surgery, Changzheng Hospital, Naval Medical University, Shanghai, 200433 China

**Keywords:** Log odds of positive lymph nodes (LODDS), Non-small cell lung cancer (NSCLC), Prognosis, Surveillance, epidemiology, and end results (SEER), Neoadjuvant therapy

## Abstract

**Background:**

Log odds of positive lymph nodes (LODDS) is a novel lymph node (LN) descriptor that demonstrates promising prognostic value in many tumors. However, there is limited information regarding LODDS in patients with non-small cell lung cancer (NSCLC), especially those receiving neoadjuvant therapy followed by lung surgery.

**Methods:**

A total of 2059 patients with NSCLC who received neoadjuvant therapy and surgery were identified from the Surveillance, Epidemiology, and End Results (SEER) database. We used the X-tile software to calculate the LODDS cutoff value. Kaplan–Meier survival analysis and receiver operating characteristic (ROC) curve analysis were performed to compare predictive values of the American Joint Committee on Cancer (AJCC) N staging descriptor and LODDS. Univariate and multivariate Cox regression and inverse probability of treatment weighting (IPTW) analyses were conducted to construct a model for predicting prognosis.

**Results:**

According to the survival analysis, LODDS had better differentiating ability than the N staging descriptor (log-rank test, *P* < 0.0001 vs. *P* = 0.031). The ROC curve demonstrated that the AUC of LODDS was significantly higher than that of the N staging descriptor in the 1-, 3-, and 5-year survival analyses (all *P* < 0.05). Univariate and multivariate Cox regression analyses showed that LODDS was an independent risk factor for patients with NSCLC receiving neoadjuvant therapy followed by surgery both before and after IPTW (all *P* < 0.001). A clinicopathological model with LODDS, age, sex, T stage, and radiotherapy could better predict prognosis.

**Conclusions:**

Compared with the AJCC N staging descriptor, LODDS exhibited better predictive ability for patients with NSCLC receiving neoadjuvant therapy followed by surgery. A multivariate clinicopathological model with LODDS demonstrated a sound performance in predicting prognosis.

**Supplementary Information:**

The online version contains supplementary material available at 10.1186/s12885-022-09908-3.

## Introduction

Lung cancer is the most common cause of cancer-related death worldwide, causing 69,410 male deaths and 62,470 female deaths in the United States alone in 2021 [1]. As a prominent type of lung cancer, non-small cell lung cancer (NSCLC) accounts for approximately 85% of all types of lung cancer, with lung adenocarcinoma and lung squamous cell carcinoma (SCC) accounting for 60 and 15% of histological subtypes, respectively [2]. With the advent of the new era of targeted therapy and immunotherapy, the overall survival (OS) of patients with NSCLC has considerably increased for each tumor stage [3]. Despite these novel treatments, lung surgery remains the most substantial and supportive tool for treating NSCLC. For patients with locally advanced NSCLC, neoadjuvant therapy plays a crucial role in downstaging lung cancer and providing an opportunity for surgery, which effectively improves prognosis [4]. Traditional neoadjuvant therapy includes chemotherapy and chemoradiation, and molecular-targeted therapy and immunotherapy are evolving as revolutionary neoadjuvant treatments for NSCLC [5]. However, tools and predictive models for predicting the prognosis of patients receiving neoadjuvant therapy followed by lung surgery are limited.

The American Joint Committee on Cancer (AJCC) TNM staging system is the most commonly used tool for predicting recurrence and survival [6]. For the N descriptor, the lymph node (LN) is based on the lymphatic region involved without any information of the number of dissected LNs (NDLN) and the number of positive LNs (NPLN) [7]. The log odds of positive LNs (LODDS) is a novel LN descriptor that has advantages over the N stating descriptor of the TNM system in many malignancies, including rectal cancer [8], gallbladder cancer [9], gastric adenocarcinoma [10], cervical cancer [11], and esophageal carcinoma [12]. LODDS is calculated using the following formula: ln([NPLN + 0.5]/[NDLN-NPLN + 0.5]). Therefore, it is usually a negative number. The higher the LODDS, the higher the NPLN, and the worse the prognosis. The LN ratio (LNR) is another N descriptor that represents the NPLN/NDLN ratio. Wang et al. reported that the nomogram combining TNM staging with LODDS+LNR performed better than the AJCC 8th TNM staging in clinical practicability [13]. Yu et al. found that LODDS exhibited better predictive power than the N, NPLN, and LNR staging systems [14]. However, no previous reports have assessed the application of LODDS in predicting the prognosis of patients receiving neoadjuvant therapy followed by lung surgery. Thus, in this study, we screened suitable cases from the SEER database and compared the value of LODDS and TNM N descriptors. Finally, we constructed a model combining LODDS with clinicopathological characteristics for better prediction. This study was conducted according to the TRIPOD reporting checklist [15].

## Materials and methods

### Patient selection

**Fig. 1:**
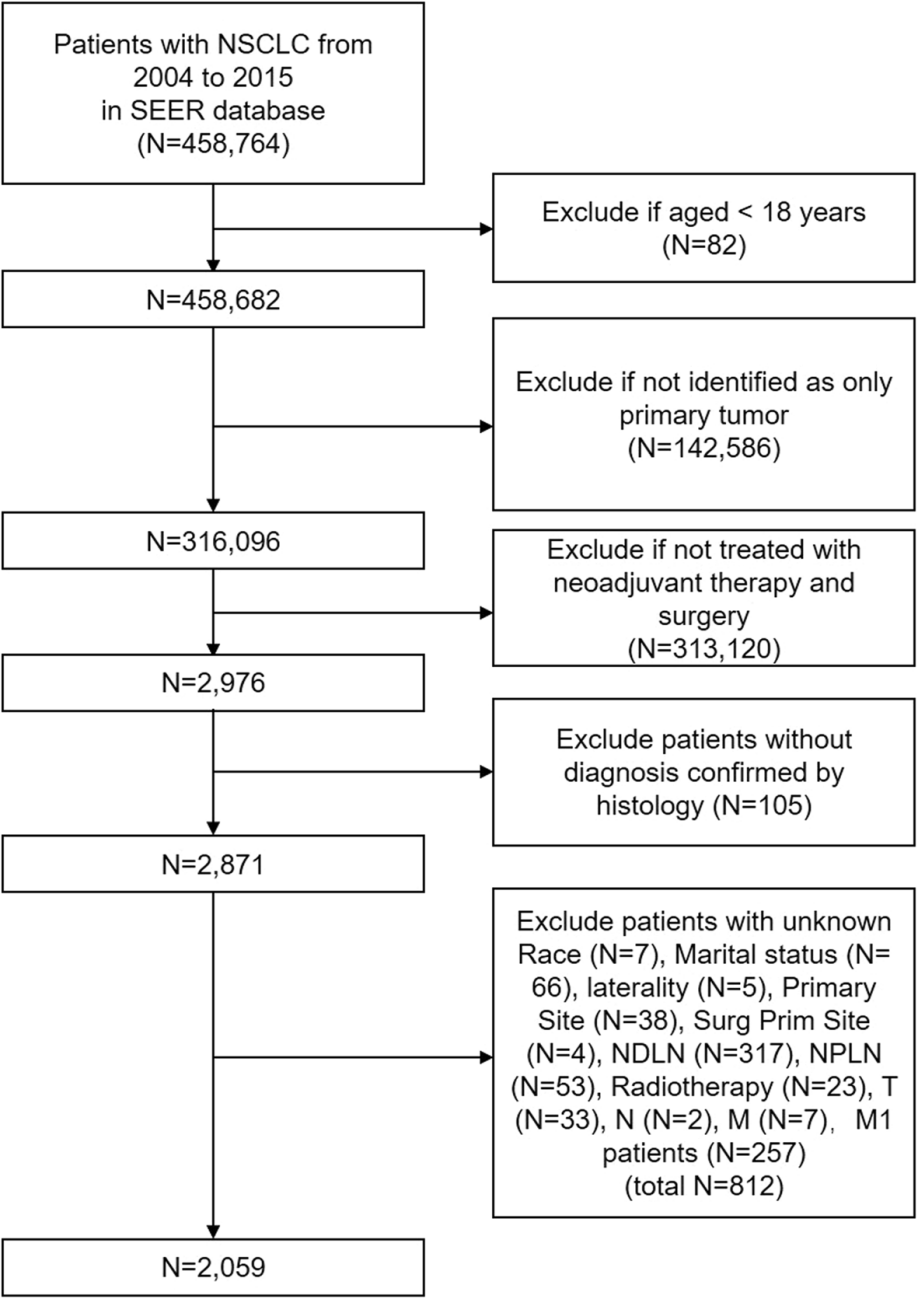
Flowchart of patient selection

All patients were selected from the SEER database (http://seer.cancer.gov/). Eighteen population-based cancer registries were selected from the SEER database, and the SEER*Stat program (v. 8.3.9) was used to extract information of patients with lung cancer. The extraction terms were as follows: “the location of the disease: lung and bronchus” and “diagnosis year: 2004–2015.” In this study, we enrolled patients with primary lung cancer who received neoadjuvant therapy and lung surgery between 2004 and 2015 (only neoadjuvant chemotherapy or radiotherapy, without any patients receiving immune checkpoint inhibitors and tyrosine kinase inhibitors). Figure 1 shows a flowchart of patient selection. The following variables were extracted: “Age recode with <1 year olds,” “Race recode (White, Black, Other),” “Sex,” “Marital status,” “Derived AJCC T, 6th ed (2004-2015),” “Derived AJCC M, 6th ed (2004-2015),” “Primary Site – labeled,” “Histologic Type ICD-O-3,” “RX Summ--Surg Prim Site (1998+),” “CS tumor size (2004-2015),” “CS Tumor Size/Ext Eval (2004-2015),” “Grade (thru 2017),” “Survival months,” “Vital status recode (study cut-off used),” “Regional nodes positive (1988+),” “Regional nodes examined (1988+),” “CS Reg Node Eval (2004-2015),” “First malignant primary indicator.” The AJCC TNM staging system was updated to the 8th version. Variables of “CS Tumor Size/Ext Eval (2004-2015)” and “CS Reg Node Eval (2004-2015)” were used to identify patients who underwent neoadjuvant therapy. The following patients were excluded: (a) patients with metastatic disease; (b) patients who did not undergo lung surgery; (c) patients in whom lung cancer was not the only primary tumor; (d) patients not receiving neoadjuvant therapy; (e) patients without information about the number of retrieved and positive LNs; and (f) patients with unknown race, marital status, tumor site, laterality, grade, T stage, and N stage.

### Ethical statement

Informed consent was waived, and ethical approval from the institutional review board was not needed because SEER is a public database and the SEER data contained no personal identifying information. This study was conducted according to the Declaration of Helsinki and the Harmonized Tripartite Guideline for Good Clinical Practice of the International Conference on Harmonization.

### LODDS calculation

LODDS was calculated using the following formula: lg([NPLN + 0.5]/[NDLN-NPLN + 0.5]), where NPLN is the number of positive LNs and NDLN is the number of dissected LNs. X-Tile software (version 3.6.1; Yale University School of Medicine, New Haven, CT, USA) was used to identify the optimal LODDS cutoff value with the maximal survival difference or highest log-rank χ^2^ value among the three groups [16]. As the X-tile software presented − 1.07 and − 0.27 as the LODDS cutoff value for the included patients, LODDS was divided into three ranges: LODDS<− 1.07, − 1.07 ≤ LODDS<-0.27, and LODDS≥-0.27.

### Statistical analysis

R software (version 4.0.2) was used for statistical analysis. Statistical significance was set at *p* values < 0.05. Categorical variables are presented as proportions. Chi-square tests or Fisher’s precision probability tests were performed for different evaluations of categorical variables. Univariate and multivariate Cox regression analyses were conducted to screen risk factors for OS when variables with *P* values < 0.05 were finally incorporated into the risk model.

Kaplan–Meier survival curves and log-rank tests were used to compare the OS of patients with different LODDS ranges and N classifications. Receiver operating characteristic (ROC) curves were used to evaluate the predictive value of the N classification, LODDS, and multivariate model for patients’ long-term outcomes. Weighted mean rank statistics were used to compare the area under the curve (AUC) of the N classification, LODDS, and multivariate model [17]. To better balance the baseline of patients with different LODDS ranges, propensity scores were determined using generalized boosted models, and inverse probability of treatment weighting (IPTW) was used to adjust the Cox regression analyses [18]. In addition, prediction accuracy was compared by calculating the integrated discrimination improvement (IDI) and net reclassification improvement (NRI) between the LODDS, N classification, and multivariate model.

## Results

### Demographic and clinicopathological characteristics

In Table 1, we compared demographic and clinicopathological characteristics of patients with different LODDS ranges. A total of 2059 patients from the SEER database were enrolled in this study and divided into three groups: LODDS<− 1.07, − 1.07 ≤ LODDS<-0.27, and LODDS≥-0.27. There was no significant difference among the groups in terms of age, marital status, surgery type, and radiotherapy (all *P* > 0.05). However, the variables of sex, race, laterality, primary site, histologic type, differentiation, and chemotherapy were significantly different among the three groups (all *P* < 0.05). Patients with LODDS≥-0.27 had higher proportions of females, right laterality, primary site of the lower lobe, adenocarcinoma, low differentiation grade, low T1 stage, and chemotherapy. Since LODDS was calculated using NDLN and NPLN, patients with LODDS≥-0.27 had a higher N stage, more regional nodes examined, and positive. We conducted IPTW to eliminate demographic and clinicopathological characteristics of patients with different LODDS ranges. As shown in Fig. S1, the absolute standardized differences in variables decreased under 0.2 and mostly under 0.1, indicating that the three groups were well matched after IPTW.

**Table 1:** Baseline characteristics of patients with NSCLC who received neoadjuvant therapy

Variables	LODDS<-1.07	-1.07 ≤ LODDS<-0.27	LODDS≥-0.27	*P*
	*n =* 866	*n =* 895	*n =* 298	
Age				0.359
≤ 60 years old	365 (42.1%)	405 (45.3%)	142 (47.7%)	
61-67 years old	218 (25.2%)	229 (25.6%)	70 (23.5%)	
≥ 68 years old	283 (32.7%)	261 (29.2%)	86 (28.9%)	
Gender				0.003
Female	381 (44.0%)	393 (43.9%)	163 (54.7%)	
Male	485 (56.0%)	502 (56.1%)	135 (45.3%)	
Race				0.029
White	729 (84.2%)	740 (82.7%)	243 (81.5%)	
Black	85 (9.8%)	84 (9.4%)	21 (7.0%)	
Other	52 (6.0%)	71 (7.9%)	34 (11.4%)	
Marital status				0.65
Married	543 (62.7%)	588 (65.7%)	189 (63.4%)	
Unmarried	121 (14.0%)	107 (12.0%)	37 (12.4%)	
Separated/Divorced/Widowed	202 (23.3%)	200 (22.3%)	72 (24.2%)	
Laterality				< 0.001
Right	481 (55.5%)	548 (61.2%)	207 (69.5%)	
Left	385 (44.5%)	347 (38.8%)	91 (30.5%)	
Primary site				< 0.001
Main bronchus	24 (2.8%)	19 (2.1%)	6 (2.0%)	
Upper lobe	642 (74.1%)	584 (65.3%)	181 (60.7%)	
Middle lobe	22 (2.5%)	41 (4.6%)	20 (6.7%)	
Lower lobe	162 (18.7%)	227 (25.4%)	83 (27.9%)	
Overlapping lesion of lung	16 (1.8%)	24 (2.7%)	8 (2.7%)	
Histologic type				< 0.001
Adenocarcinoma	343 (39.6%)	451 (50.4%)	205 (68.8%)	
Squamous cell	365 (42.1%)	291 (32.5%)	57 (19.1%)	
Other	158 (18.2%)	153 (17.1%)	36 (12.1%)	
Differentiation				0.032
Grade I	32 (3.7%)	29 (3.2%)	9 (3.0%)	
Grade II	224 (25.9%)	238 (26.6%)	101 (33.9%)	
Grade III	403 (46.5%)	417 (46.6%)	138 (46.3%)	
Grade IV	33 (3.8%)	23 (2.6%)	2 (0.7%)	
Unknown	174 (20.1%)	188 (21.0%)	48 (16.1%)	
T				< 0.001
T1	91 (10.5%)	147 (16.4%)	58 (19.5%)	
T2	208 (24.0%)	257 (28.7%)	129 (43.3%)	
T3	279 (32.2%)	270 (30.2%)	67 (22.5%)	
T4	288 (33.3%)	221 (24.7%)	44 (14.8%)	
N				
N0	447 (51.6%)	178 (19.9%)	0 (0.0%)	< 0.001
N1	112 (12.9%)	198 (22.1%)	42 (14.1%)	
N2	293 (33.8%)	502 (56.1%)	246 (82.6%)	
N3	14 (1.6%)	17 (1.9%)	10 (3.4%)	
Regional nodes examined	13.0 (9.0-20.0)	9.0 (4.0-15.0)	9.0 (5.0-15.0)	< 0.001
Regional nodes positive	.0 (0.0-0.0)	1.0 (0.0-2.0)	5.0 (2.0-9.0)	< 0.001
Surgery				0.418
Sublobectomy	23 (2.7%)	39 (4.4%)	11 (3.7%)	
Lobectomy	676 (78.1%)	691 (77.2%)	233 (78.2%)	
Pneumonectomy	167 (19.3%)	165 (18.4%)	54 (18.1%)	
Radiotherapy				0.082
No/Unknown	291 (33.6%)	275 (30.7%)	80 (26.8%)	
Yes	575 (66.4%)	620 (69.3%)	218 (73.2%)	
Chemotherapy				0.008
No/Unknown	24 (2.8%)	15 (1.7%)	0 (0.0%)	
Yes	842 (97.2%)	880 (98.3%)	298 (100.0%)	

Categorical variables are presented as numbers (percentages), and continuous variables are reported as medians with interquartile ranges. *NSCLC* Non-small cell lung cancer, *LODDS* Log odds of positive lymph nodes

### Univariate and multivariate cox regression analyses

We conducted univariate and multivariate Cox regression analyses to confirm independent risk factors for patient survival, as shown in Tables 2 and 3. Before IPTW, univariate analysis demonstrated that LODDS, age, sex, T stage, N stage, and radiotherapy were significantly associated with OS (all *P* < 0.05). However, multivariate analysis showed that LODDS, age, sex, T stage, and radiotherapy were independent risk factors for patient survival (all *P* < 0.05), with N stage excluded.

**Table 2:** Cox regression analysis of patients with NSCLC who received neoadjuvant therapy before IPTW

Variables	Univariable analysis		Multivariable analysis	
	HR (95%CI)	*P*	HR (95%CI)	*P*
LODDS				
LODDS<-1.07	Reference		Reference	
-1.07 ≤ LODDS<-0.27	1.387 (1.220-1.578)	< 0.001	1.396 (1.220-1.598)	< 0.001
LODDS≥-0.27	2.026 (1.719-2.388)	< 0.001	2.116 (1.759-2.544)	< 0.001
Age				
≤60 years old	Reference		Reference	
61-67 years old	1.300 (1.123-1.504)	< 0.001	1.353 (1.168-1.568)	< 0.001
≥ 68 years old	1.583 (1.385-1.808)	< 0.001	1.716 (1.497-1.967)	< 0.001
Gender				
Female	Reference		Reference	
Male	1.233 (1.098-1.384)	< 0.001	1.246 (1.107-1.401)	< 0.001
Race				
White	Reference		N/A	
Black	0.874 (0.715-1.069)	0.19	N/A	
Other	0.858 (0.685-1.075)	0.182	N/A	
Marital status				
Married	Reference		N/A	
Unmarried	1.106 (0.929-1.316)	0.259	N/A	
Separated/Divorced/Widowed	1.064 (0.926-1.222)	0.383	N/A	
Laterality				
Right	Reference		Reference	
Left	0.900 (0.800-1.012)	0.078	0.958 (0.848-1.083)	0.495
Primary site				
Main bronchus	Reference		Reference	
Upper lobe	1.105 (0.736-1.659)	0.631	0.968 (0.642-1.460)	0.876
Middle lobe	1.436 (0.887-2.327)	0.141	1.204 (0.736-1.967)	0.46
Lower lobe	1.423 (0.939-2.158)	0.096	1.223 (0.803-1.861)	0.348
Overlapping lesion of lung	1.826 (1.083-3.079)	0.024	1.383 (0.814-2.348)	0.231
Histologic type				
Adenocarcinoma	Reference		N/A	
Squamous cell	0.986 (0.867-1.121)	0.825	N/A	
Other	0.935 (0.796-1.098)	0.411	N/A	
Differentiation				
Grade I	Reference		Reference	
Grade II	1.266 (0.888-1.803)	0.192	1.229 (0.861-1.753)	0.256
Grade III	1.403 (0.993-1.983)	0.055	1.358 (0.959-1.923)	0.085
Grade IV	1.342 (0.834-2.160)	0.225	1.431 (0.886-2.310)	0.143
Unknown	1.099 (0.764-1.580)	0.611	1.091 (0.757-1.574)	0.639
T				
T1	Reference		Reference	
T2	1.153 (0.954-1.392)	0.14	1.147 (0.948-1.389)	0.158
T3	1.277 (1.061-1.537)	0.01	1.363 (1.128-1.647)	0.001
T4	1.175 (0.969-1.424)	0.101	1.336 (1.094-1.632)	0.004
N				
N0	Reference		Reference	
N1	1.132 (0.951-1.348)	0.163	0.934 (0.778-1.120)	0.459
N2	1.223 (1.069-1.398)	0.003	1.021 (0.878-1.186)	0.791
N3	1.240 (0.819-1.877)	0.309	0.962 (0.631-1.468)	0.858
Surgery				
Sublobectomy	Reference		N/A	
Lobectomy	0.866 (0.648-1.159)	0.334	N/A	
Pneumonectomy	1.086 (0.796-1.482)	0.602	N/A	
Radiotherapy				
No/Unknown	Reference		Reference	
Yes	1.173 (1.035-1.330)	0.013	1.203 (1.054-1.372)	0.006
Chemotherapy				
No/Unknown	Reference		N/A	
Yes	0.927 (0.619-1.389)	0.715	N/A	

*NSCLC* Non-small cell lung cancer, *IPTW* Inverse probability of treatment weighting, *HR* Hazard ratio, *CI* Confidence interval, *LODDS* Log odds of positive lymph nodes

**Table 3:** Cox regression analysis of patients with NSCLC who received neoadjuvant therapy after IPTW

Variables	Univariable analysis		Multivariable analysis	
	HR (95%CI)	*P*	HR (95%CI)	*P*
LODDS				
LODDS<-1.07	Reference		Reference	
-1.07 ≤ LODDS<-0.27	1.445 (1.325-1.577)	< 0.001	1.437 (1.313-1.573)	< 0.001
LODDS≥-0.27	2.318 (2.127-2.527)	< 0.001	2.459 (2.227-2.715)	< 0.001
Age				
≤ 60 years old	Reference		Reference	
61-67 years old	1.282 (1.177-1.397)	< 0.001	1.360 (1.245-1.485)	< 0.001
≥ 68 years old	1.591 (1.471-1.721)	< 0.001	1.803 (1.659-1.959)	< 0.001
Gender				
Female	Reference		Reference	
Male	1.318 (1.231-1.411)	< 0.001	1.321 (1.227-1.422)	< 0.001
Race				
White	Reference		Reference	
Black	0.919 (0.814-1.037)	0.169	0.989 (0.874-1.119)	0.86
Other	0.827 (0.723-0.946)	0.006	0.798 (0.694-0.917)	0.001
Marital status				
Married	Reference		Reference	
Unmarried	1.194 (1.078-1.324)	< 0.001	1.417 (1.272-1.578)	< 0.001
Separated/Divorced/Widowed	1.064 (0.981-1.155)	0.135	1.163 (1.067-1.269)	< 0.001
Laterality				
Right	Reference		N/A	
Left	1.002 (0.935-1.073)	0.961	N/A	
Primary site				
Main bronchus	Reference		Reference	
Upper lobe	1.068 (0.833-1.370)	0.603	1.109 (0.857-1.434)	0.432
Middle lobe	1.130 (0.842-1.516)	0.416	1.265 (0.934-1.714)	0.128
Lower lobe	1.293 (1.002-1.668)	0.048	1.373 (1.056-1.785)	0.018
Overlapping lesion of lung	1.498 (1.079-2.078)	0.016	1.233 (0.885-1.719)	0.216
Histologic type				
Adenocarcinoma	Reference		Reference	
Squamous cell	1.111 (1.031-1.197)	0.006	1.022 (0.942-1.109)	0.598
Other	0.982 (0.893-1.081)	0.716	0.911 (0.822-1.009)	0.073
Differentiation				
Grade I	Reference		Reference	
Grade II	1.254 (1.015-1.550)	0.036	1.223 (0.986-1.516)	0.067
Grade III	1.361 (1.106-1.674)	0.004	1.258 (1.018-1.554)	0.034
Grade IV	1.153 (0.843-1.577)	0.373	1.289 (0.936-1.776)	0.12
Unknown	1.116 (0.899-1.386)	0.321	1.140 (0.913-1.423)	0.247
T				
T1	Reference		Reference	
T2	1.163 (1.038-1.302)	0.009	1.116 (0.993-1.253)	0.065
T3	1.337 (1.196-1.494)	< 0.001	1.337 (1.194-1.497)	< 0.001
T4	1.405 (1.253-1.575)	< 0.001	1.468 (1.302-1.656)	< 0.001
N				
N0	Reference		Reference	
N1	1.356 (1.219-1.508)	< 0.001	0.930 (0.829-1.043)	0.216
N2	1.416 (1.299-1.544)	< 0.001	1.046 (0.947-1.156)	0.377
N3	1.542 (1.231-1.931)	< 0.001	1.062 (0.840-1.342)	0.617
Surgery				
Sublobectomy	Reference		Reference	
Lobectomy	0.819 (0.687-0.977)	0.027	0.913 (0.762-1.093)	0.32
Pneumonectomy	1.028 (0.851-1.241)	0.776	1.156 (0.949-1.408)	0.149
Radiotherapy				
No/Unknown	Reference		Reference	
Yes	1.065 (0.989-1.146)	0.096	1.067 (0.987-1.153)	0.105
Chemotherapy				
No/Unknown	Reference		N/A	
Yes	0.936 (0.692-1.266)	0.67	N/A	

*NSCLC* Non-small cell lung cancer, *IPTW* Inverse probability of treatment weighting, *HR* Hazard ratio, *CI* Confidence interval, *LODDS* Log odds of positive lymph nodes

After IPTW, the results of the univariate analysis were similar to previous results, showing that LODDS, age, sex, T stage, N stage, and radiotherapy were statistically significant variables, whereas race, marital status, primary site, histologic type, differentiation, and surgery type were newly added variables (all *P* < 0.05). Furthermore, multivariate regression analysis indicated that LODDS, age, sex, race, marital status, primary site, differentiation, and T stage were independent risk factors for patient survival (all *P <* 0.05), with N stage excluded. With or without IPTW, LODDS was an independent risk factor for the prognosis of patients receiving neoadjuvant therapy followed by lung surgery.

We also conducted subgroup analysis to further validate the significance of LODDS. We further compared the relative risks of different LODDS ranges by dividing patients into different subgroups based on the variable. We found that a higher LODDS was associated with a higher risk in most subgroups, as shown in Table 4. However, there was no statistical significance among the different LODDS ranges with respect to middle lobe, overlapping primary site, grade I differentiation, grade IV differentiation, and N3 stage, which could be because of the relatively small sample size.

**Table 4:** Multivariable Cox regression analysis of subgroups of patients with NSCLC who received neoadjuvant therapy

Subgroups	LODDS<-1.07	-1.07 ≤ LODDS<-0.27		LODDS≥-0.27	
	HR	HR (95%CI)	P	HR (95%CI)	P
Age					
≤ 60 years old	Reference	1.649 (1.319-2.061)	< 0.001	2.587 (1.924-3.477)	< 0.001
61-67 years old	Reference	1.302 (0.985-1.721)	0.064	1.709 (1.148-2.544)	0.008
≥ 68 years old	Reference	1.299 (1.040-1.623)	0.021	2.018 (1.477-2.756)	< 0.001
Gender					
Female	Reference	1.197 (0.967-1.482)	0.098	1.893 (1.449-2.472)	< 0.001
Male	Reference	1.503 (1.261-1.791)	< 0.001	2.252 (1.739-2.917)	< 0.001
Laterality					
Right	Reference	1.260 (1.060-1.498)	0.009	1.832 (1.456-2.305)	< 0.001
Left	Reference	1.638 (1.320-2.033)	< 0.001	2.646 (1.936-3.617)	< 0.001
Primary site					
Main bronchus	Reference	1.207 (0.351-4.149)	0.766	5.019 (0.974-25.856)	0.054
Upper lobe	Reference	1.415 (1.202-1.667)	< 0.001	2.305 (1.829-2.905)	< 0.001
Middle lobe	Reference	1.853 (0.734-4.676)	0.192	1.741 (0.600-5.053)	0.308
Lower lobe	Reference	1.375 (1.037-1.822)	0.027	1.813 (1.244-2.642)	0.002
Overlapping lesion of lung	Reference	1.684 (0.562-5.046)	0.352	0.745 (0.126-4.396)	0.746
Differentiation					
Grade I	Reference	1.723 (0.680-4.362)	0.251	1.940 (0.538-6.998)	0.311
Grade II	Reference	1.361 (1.030-1.800)	0.030	2.259 (1.592-3.205)	< 0.001
Grade III	Reference	1.301 (1.077-1.571)	0.006	1.971 (1.521-2.554)	< 0.001
Grade IV	Reference	0.764 (0.338-1.728)	0.518	0.245 (0.018-3.310)	0.290
Unknown	Reference	2.298 (1.630-3.239)	< 0.001	2.923 (1.795-4.761)	< 0.001
T					
T1	Reference	1.302 (0.855-1.981)	0.219	1.821 (1.071-3.096)	0.027
T2	Reference	1.582 (1.193-2.098)	0.001	2.003 (1.444-2.778)	< 0.001
T3	Reference	1.468 (1.165-1.850)	0.001	2.115 (1.482-3.017)	< 0.001
T4	Reference	1.184 (0.924-1.516)	0.182	2.291 (1.510-3.476)	< 0.001
N					
N0	Reference	0.987 (0.778-1.251)	0.911	NA	NA
N1	Reference	1.393 (0.993-1.953)	0.055	1.939 (1.221-3.078)	0.005
N2	Reference	1.786 (1.444-2.209)	< 0.001	2.523 (1.983-3.211)	< 0.001
N3	Reference	5.538 (1.011-30.326)	0.049	3.885 (0.377-40.074)	0.254
Radiotherapy					
No/Unknown	Reference	1.532 (1.190-1.972)	0.001	2.901 (2.052-4.101)	< 0.001
Yes	Reference	1.348 (1.149-1.582)	< 0.001	1.884 (1.513-2.346)	< 0.001

HRs of multivariate analysis of subgroups were adjusted for age, sex, laterality, primary site, differentiation, T stage, N stage, and radiotherapy, except for the subgroup variable itself. LODDS, log odds of positive lymph nodes; NSCLC, non-small cell lung cancer; HR, hazard ratio; CI, confidence interval

### Survival analysis

**Fig. 2:**
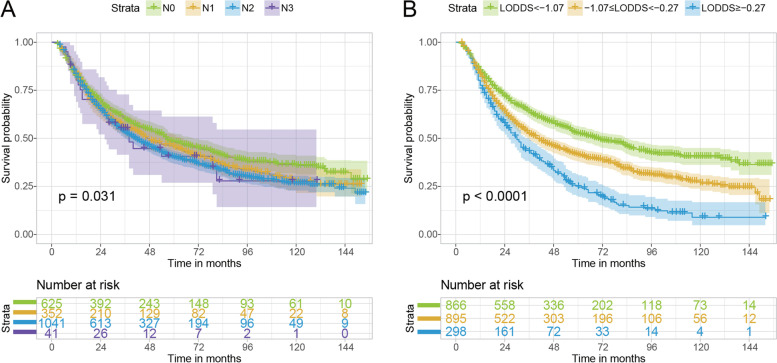
Kaplan–Meier estimates of OS of patients with NSCLC who received neoadjuvant therapy stratified by N classification (**A**) and LODDS (**B**) before IPTW. OS, overall survival; NSCLC, non-small cell lung cancer; LODDS, log odds of positive lymph node; IPTW, inverse probability of treatment weighting

**Fig. 3:**
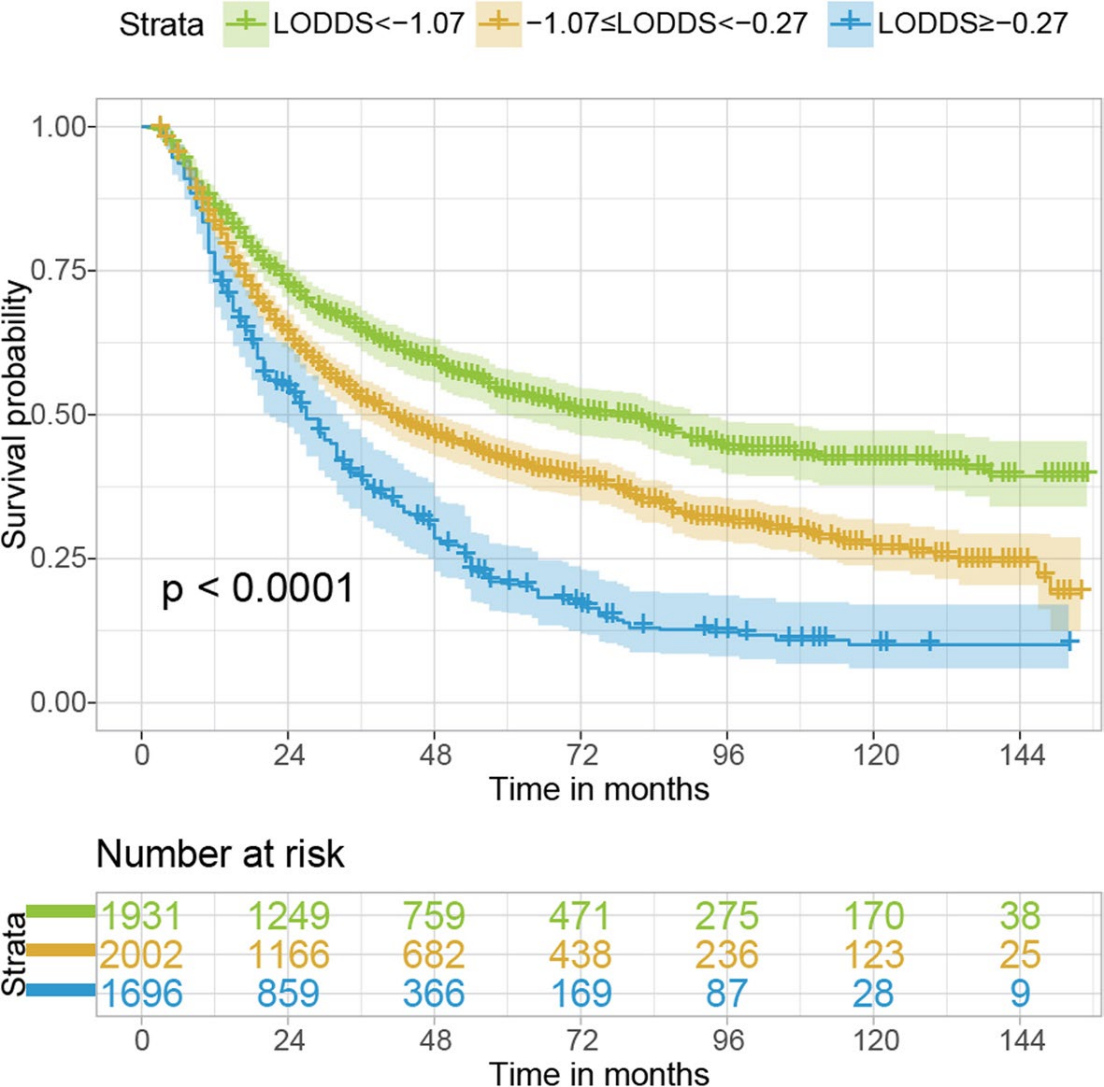
Kaplan–Meier estimates of OS of patients with NSCLC who received neoadjuvant therapy stratified by LODDS after IPTW. OS, overall survival; NSCLC, non-small cell lung cancer; LODDS, log odds of positive lymph node; IPTW, inverse probability of treatment weighting

We compared the long-term survival of patients with different N classifications (Fig. 2A). Although patients with different N stages presented different survival curves with *P* values of 0.036, the curve was not separate and mostly overlapped. Nevertheless, when we divided patients into three groups based on LODDS ranges, we found that the curve was much more distinct (Fig. 2B). Patients with LODDS<-1.07 had the best survival status compared to patients in the other two groups, while patients in the middle group (− 1.07 ≤ LODDS<-0.27) had better OS than those with LODDS≥-0.27 (*P* < 0.0001). Even after IPTW, the survival curve remained significant among the three groups (*P <* 0.0001), as shown in Fig. 3.

### ROC curve analysis

**Fig. 4:**
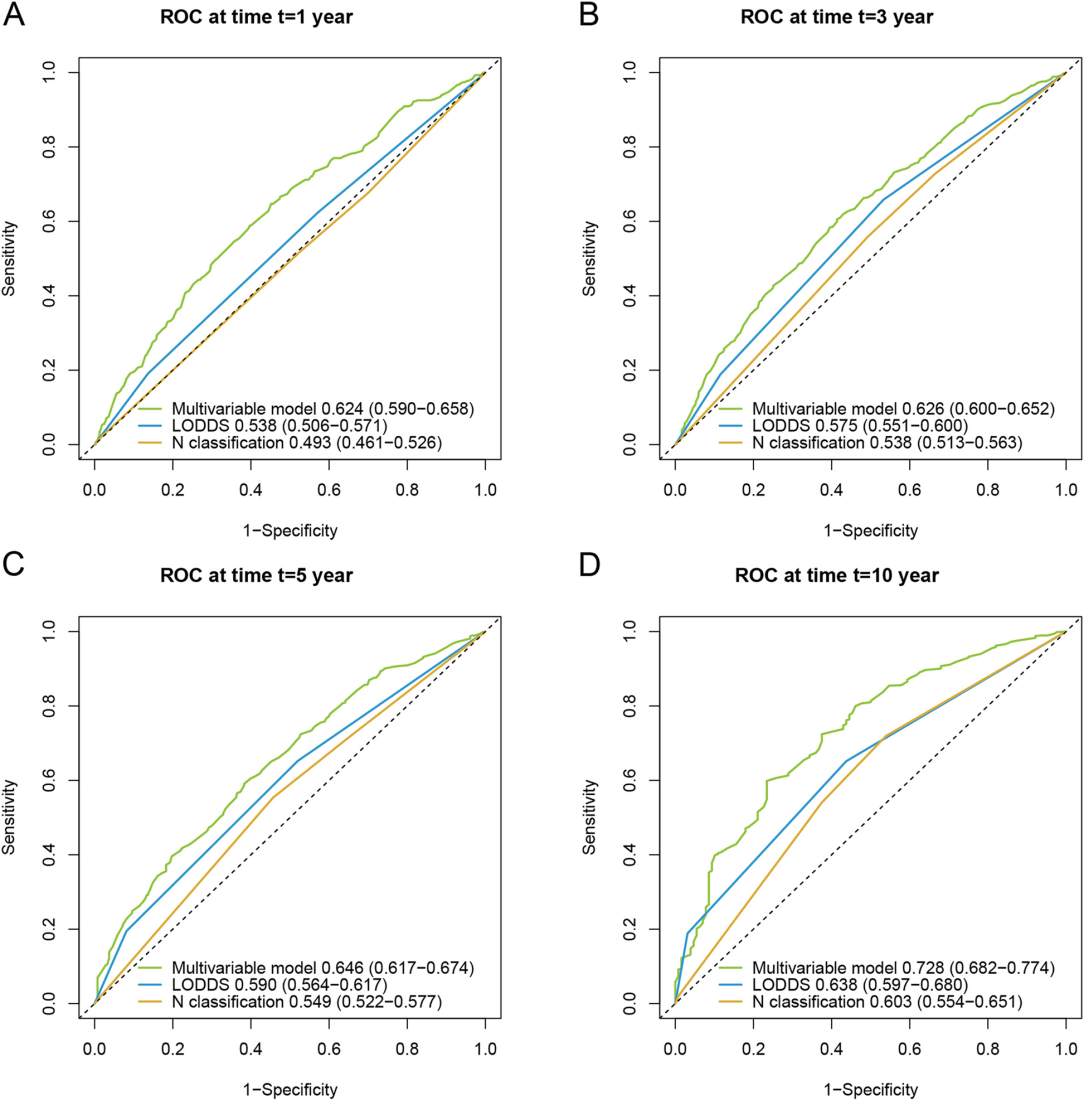
ROC curves for a multivariable model (including LODDS, age, sex, T stage, and radiotherapy), LODDS, and N classification predicting 1-year (**A**), 3-year (**B**), 5-year (**C**), and 10-year (**D**) OS of patients with NSCLC who received neoadjuvant therapy. ROC, receiver operating characteristic; LODDS, log odds of positive lymph node; OS, overall survival; NSCLC, non-small cell lung cancer

We compared the accuracy and prognostic value of the N classification, LODDS, and multivariate model using ROC curves and AUC comparisons. We used a multivariate model with five variables that were independent prognostic indicators in the multivariate analysis in Table 2: LODDS, age, sex, T stage, and radiotherapy. As shown in Fig. 4, LODDS had a significantly higher AUC than the N classification for 1-year (*P* = 0.008), 3-year (*P* = 0.007), and 5-year OS (*P* = 0.010) but not at 10-year OS (*P* = 0.228). However, the multivariate model had a significantly higher AUC than LODDS and N classification for 1-, 3-, 5-, and 10-year OS (all *P* < 0.001). We also compared the IDI and NRI of the N classification, LODDS, and multivariate model, as shown in Table 5. On considering LODDS as a reference, we found that the IDI and NRI of the N classification were negative. At the same time, those of the multivariate model were positive, suggesting that the LODDS had significantly higher predictive accuracy than the N classification but had lower predictive accuracy than the multivariate model (*P* < 0.05).

**Table 5:** Comparison of predictive performance between LODDS and other models

Model	IDI (95%CI)	*P*	NRI (95%CI)	*P*
1-year OS				
LODDS	Reference		Reference	
N classification	−0.007 (− 0.014 to − 0.002)	0.007	− 0.060 (− 0.122 to 0.002)	0.060
Multivariable model	0.020 (0.012 to 0.031)	< 0.001	0.184 (0.114 to 0.250)	< 0.001
3-year OS				
LODDS	Reference		Reference	
N classification	−0.018 (− 0.030 to − 0.007)	< 0.001	− 0.049 (− 0.157 to − 0.001)	0.047
Multivariable model	0.029 (0.017 to 0.045)	< 0.001	0.136 (0.096 to 0.196)	< 0.001
5-year OS				
LODDS	Reference		Reference	
N classification	−0.025 (− 0.040 to − 0.012)	< 0.001	− 0.074 (− 0.183 to − 0.025)	0.007
Multivariable model	0.036 (0.024 to 0.057)	< 0.001	0.171 (0.126 to 0.224)	< 0.001
10-year OS				
LODDS	Reference		Reference	
N classification	−0.030 (− 0.050 to − 0.010)	< 0.001	−0.200 (− 0.290 to − 0.006)	0.027
Multivariable model	0.056 (0.034 to 0.086)	< 0.001	0.258 (0.168 to 0.348)	< 0.001

The multivariable model included LODDS, age, sex, T stage, and radiotherapy. LODDS, log odds of positive lymph nodes; OS, overall survival; IDI, integrated discrimination improvement; NRI, net reclassification improvement; CI, confidence interval

## Discussion

Controversies regarding the nodal status of the 8th TNM staging system have existed for several years. In summary, there are four commonly used nodal classifications for lung cancer: N classification, NPLN, LNR, and LODDS [19]. The N classification in the TNM staging system is the most commonly used prognostic tool for patients with lung cancer. The N classification for lung cancer is easy to understand and remember; it categorizes no metastasis to LNs as N0, metastasis to ipsilateral peribronchial and/or hilar nodes and intrapulmonary nodes as N1, metastasis to ipsilateral mediastinal and/or subcarinal nodes as N2, and metastasis to contralateral mediastinal and/or hilar nodes and any supraclavicular LNs as N3 [20]. The TNM staging system helps clinicians determine treatment and predict prognosis. However, the N classification is based on the anatomic position of positive nodes, without any quantitative information, leading to inaccuracy and low discrimination power [21]. In this study, we found that the AUCs of the N classification were 0.493 (95% CI 0.461–0.526), 0.538 (95% CI 0.513–0.563), 0.549 (95% CI 0.522–0.577), and 0.603 (95% CI 0.554–0.651) for 1-, 3-, 5-, and 10-year survival, respectively. The low discriminative power of the N classification of the TNM staging calls for a more accurate nodal status assessment tool.

For patients undergoing radical lung cancer resection, systematic LN dissection (SND) is the standard procedure for surgical treatment of NSCLC [22], especially for patients receiving neoadjuvant therapy who are usually diagnosed with stage II–III NSCLC, when systematic LN dissection is necessary. In this study, 78.1% of patients underwent lobectomy and 19.3% underwent pneumonectomy, with only 2.7% of patients undergoing sublobectomy. Mun et al. reported that lobe-specific mediastinal LN dissection is vital for patients with pN1, whereas SND contributes to survival in patients with pN1 after recurrence [23]. The LNs retrieved during surgery provide sufficient knowledge about nodal status with quantitative information. NPLN represents the number of positive LNs requiring retrieval of LNs during surgery [14]. However, NPLN can be significantly affected by the surgical technique and number of examined LNs because the pathological results are dependent on LN dissection. Using a Chinese multi-institutional registry and the US SEER database for stage I–IIIA resected NSCLC, Liang et al. recommended that 16 LNs should be examined for prognostic stratification [24].

Ratio-based nodal evaluation methods are also used and do not require information of the number of examined LNs, including LNR and LODDS. LNR is calculated by dividing NPLN with NDLN. LODDS is calculated using the formula: log (NPLN+ 0.50)/(NDLN−NPLN+ 0.50). Therefore, LODDS is the only indicator that includes the numbers of dissected, positive, and negative LNs. The controversy regarding the comparison between LNR and LODDS is that they demonstrate advantages in different situations [25, 26]. However, LODDS was superior to LNR for lung cancer in most studies. Yu et al. demonstrated that LODDS showed better predictive performance than the N classification, NPLN, and LNR in patients with node-positive SCC after surgery [14]. Deng et al. found that LODDS and LNR performed slightly differently in patients with different resected LNs. They proved that LODDS was slightly better than LNR for patients with < 10 resected LNs, whereas LNR was slightly better than LODDS for patients with ≥10 resected LNs [27]. When combined, LODDS and LNR had the highest predictive accuracy compared with other models for cancer-specific survival and OS of patients with lung adenocarcinoma after surgery [13]. However, there are no previous reports on the predictive ability and accuracy of LODDS in patients receiving neoadjuvant therapy and surgery. In this study, we found that LODDS could effectively differentiate patients’ prognoses. In addition, LODDS demonstrated a much higher AUC than N classification for 1-, 3-, and 5-year OS prediction but not for 10-year OS prediction. Univariate and multivariate Cox regression analyses demonstrated that LODDS was an independent risk factor for patients’ OS. Subgroup analyses confirmed the results in the different subgroups.

We noticed that baseline characteristics and demographic data of patients with different LODDS ranges were significantly different. To eliminate the bias caused by this difference, we applied IPTW to balance the baseline characteristics and demographic data. With or without IPTW, LODDS showed statistical significance in the Kaplan–Meier curve and regression analyses. Because of its excellent predictive ability, LODDS was incorporated into the multivariate model to construct a nomogram. Wang’s nomogram included LODDS+LNR as the nodal status factor and showed excellent predictive ability with a high C-index (0.7222 for the CSS nomogram, 0.6920 for the OS nomogram) for patients with T1-4N0-2M0 lung adenocarcinoma after surgery [13]. This study used a multivariate model with five critical factors: LODDS, age, sex, T stage, and radiotherapy. The model showed a higher AUC than the N classification and LODDS. The multivariate model’s predictive performance indicators, IDI and NRI, were also higher than those of the N classification and LODDS, which proved that LODDS is an independent and compatible factor for LN staging and could be incorporated into the risk assessment model well.

Compared with the N descriptor, NPLN, LNR, and LODDS had an unignorable shortcoming. They depended on the dissection of LNs and pathological results, while the N descriptor could be determined using PET-CT and LN biopsy. Therefore, the N stage can directly decide the TNM stage and the following treatment approach before surgery; however, NPLN, LNR, and LODDS can only be adopted as tools to predict recurrence and prognosis after surgery. This study had several limitations. On the one hand, many important data are absent in the SEER database, including smoking history, sequence of surgery and chemotherapy, and novel treatments with tyrosine kinase inhibitors and immune checkpoint inhibitors. Missing data may lead to a worse predictive effect of the nomogram. We attempted to construct a nomogram based on our findings but failed in this study because the C-index was very low. We suspected that the low C-index of the nomogram was because of the heterogeneity of patients who received very different treatment regimens. On the other hand, the new era of tyrosine kinase inhibitors and immune checkpoint inhibitors brings a paradigm shift for neoadjuvant therapy for patients with NSCLC, which challenges LODDS and other nodal status indicators.

## Conclusions

For patients with NSCLC receiving neoadjuvant therapy followed by surgery, LODDS had better predictive ability than the AJCC N staging descriptor. A multivariate clinicopathological model with LODDS demonstrated excellent performance in predicting prognosis. LODDS provides clinicians with more accurate nodal status information, while nomograms and external validation are required in future studies.

## Supplementary Information

**Additional file 1: Fig. S1.** Absolute standard difference in covariables (age, sex, race, marital status, laterality, primary site, histologic type, differentiation, T stage, N stage, surgery, radiotherapy, and chemotherapy) between subgroups of LODDS before and after IPTW. Blue lines indicate a reduction, while red lines indicate an increase in absolute standard difference. Closed red circles indicate a statistically significant difference, and hollow red circles indicate a not statistically significant difference. IPTW, inverse probability of treatment weighting; LODDS, log odds of positive lymph node.

## Data Availability

The dataset supporting the conclusions of this article is available in the SEER*Stat software (version 8.3.8; RRID:SCR_003293; https://seer.cancer.gov/resources/). The primary data can be accessed through SEER*Stat software with certain filter according to the methods in the manuscript.
