# Supplementary material for: Evaluation of log odds of positive lymph nodes in predicting the survival of patients with non-small cell lung cancer treated with neoadjuvant therapy and surgery: a SEER cohort-based study
Source: BMC Cancer. 2022 Jul 20;22:801. doi: 10.1186/s12885-022-09908-3 (PMC9297565; doi:10.1186/s12885-022-09908-3)
Supplement: Supplementary file 1 — Additional file 1: Fig. S1. Absolute standard difference in covariables (age, sex, race, marital status, laterality, primary site, histologic type, differentiation, T stage, N stage, surgery, radiotherapy, and chemotherapy) between subgroups of LODDS before and after IPTW. Blue lines indicate a reduction, while red lines indicate an increase in absolute standard difference. Closed red circles indicate a statistically significant difference, and hollow red circles indicate a not statistically significant difference. IPTW, inverse probability of treatment weighting; LODDS, log odds of positive lymph node. [file 12885_2022_9908_MOESM1_ESM.docx]

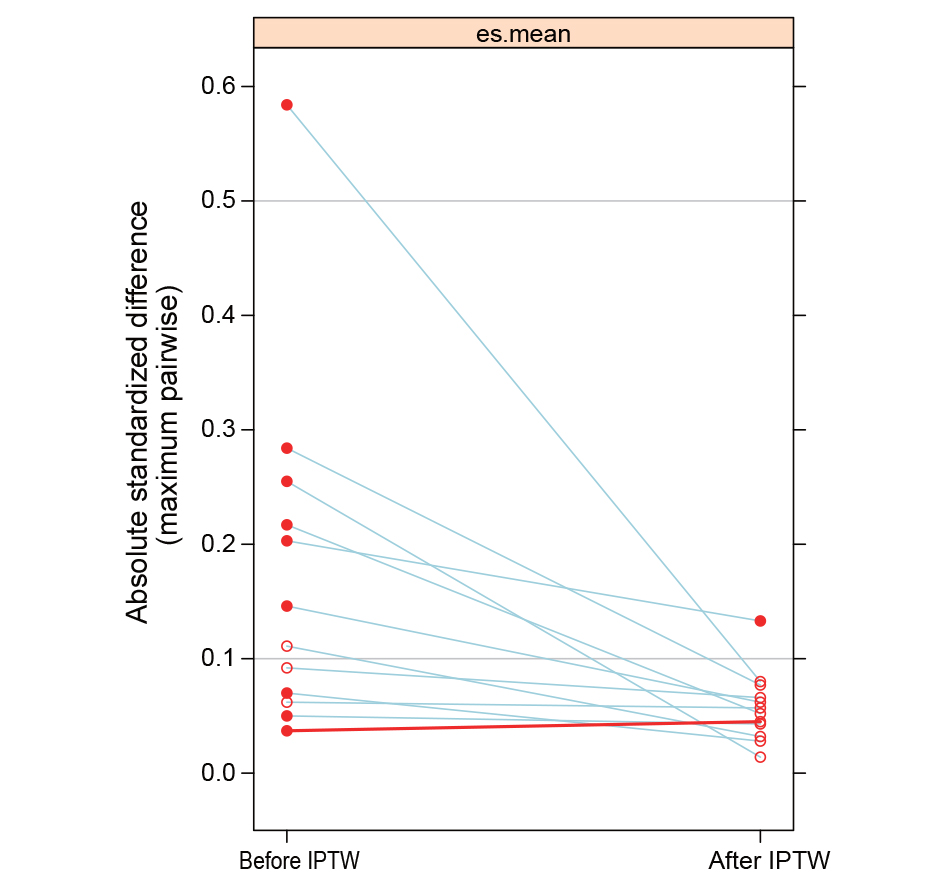


**FIGURE S1 Absolute standard difference of covariables (age, gender, race, marital status, laterality, primary site, histologic type, differentiation, T, N, surgery, radiotherapy, and chemotherapy) between subgroups of LODDS before and after IPTW.** Blue lines mean a reduction, while red lines mean an increase in absolute standard difference. Closed red circles indicate a statistically significant difference, and hollow red circles indicate a not statistically significant difference. IPTW, inverse probability of treatment weighting; LODDS, log odds of the positive lymph node.
